# Performance of the pattern‐based interpretation of p53 immunohistochemistry as a surrogate for *TP53* mutations in vulvar squamous cell carcinoma

**DOI:** 10.1111/his.14109

**Published:** 2020-06-07

**Authors:** Kim E Kortekaas, Nienke Solleveld‐Westerink, Basile Tessier‐Cloutier, Tessa A Rutten, Mariëtte I E Poelgeest, C Blake Gilks, Lien N Hoang, Tjalling Bosse

**Affiliations:** ^1^ Department of Obstetrics & Gynecology Leiden University Medical Center Leiden the Netherlands; ^2^ Department of Pathology Leiden University Medical Center Leiden the Netherlands; ^3^ Department of Pathology and Laboratory Medicine Vancouver General Hospital Vancouver BC Canada

**Keywords:** p53 immunohistochemistry, squamous cell carcinoma, surrogate marker, *TP53* mutations, vulvar cancer

## Abstract

**Aims:**

The most commonly mutated gene in vulvar squamous cell carcinoma (VSCC) is *TP53* and its prognostic value, particularly in HPV‐independent VSCC, is uncertain. In other tumours, p53 immunohistochemistry (IHC) is an excellent surrogate marker for *TP53* mutations. In order to study this in VSCC, we assigned six p53 IHC patterns into two final classes: ‘wild‐type’ or ‘mutant’. We determined the performance and interobserver variability of this pattern‐based p53 IHC approach.

**Methods and results:**

Two experienced gynaecological pathologists scored the predefined p53 IHC patterns of 59 VSCC, independently and blinded for molecular data. Agreement was calculated by Cohen's kappa. All disagreements regarding p53 IHC patterns were resolved by a consensus meeting. After DNA isolation, the presence of pathogenic *TP53* variants was determined by next‐generation sequencing (NGS). Sensitivity, specificity and accuracy of p53 IHC as a surrogate marker for *TP53* mutation status were calculated. Initial p53 IHC pattern interpretation showed substantial agreement between both observers (*k* = 0.71, *P* < 0.001). After consensus, 18 cases (30.5%) were assigned a final p53 IHC class as *TP53* wild‐type and 41 cases (69.5%) as mutant. The accuracy between the p53 IHC class and *TP53* mutation status, after the consensus meeting, was 96.6%. Moreover, the sensitivity and specificity were high 95.3% [95% confidence interval (CI) = 82.9–99.1% and 100% (95% CI = 75.9–100%)].

**Conclusions:**

Pattern‐based p53 IHC classification is highly reproducible among experienced gynaecological pathologists and accurately reflects *TP53* mutations in VSCC. This approach to p53 IHC interpretation offers guidance and provides necessary clarity for resolving the proposed prognostic relevance of final p53 IHC class within HPV‐independent VSCC.

## Introduction

Molecular testing is rapidly being introduced into the classification systems of many malignancies throughout pathology, including gynaecological pathology. These integrated molecular classification systems result in biologically homogeneous ‘histo‐molecular’ entities that are well‐suited for future trial designs in which novel (targeted) treatments can be tested. To facilitate the rapid implementation of these novel approaches in diagnostic pathology, reliable surrogate markers are required. For vulvar squamous cell carcinoma (VSCC) it has long been recognised that at least two ‘histo‐molecular’ subclasses can be recognized: HPV (human papillomavirus)‐associated VSCC and VSCC independent of HPV.^1^, ^2^ In order to separate these two VSCC subtypes, p16 immunohistochemistry (IHC) has been shown to be a reliable surrogate marker (sensitivity = 100%, specificity = 98.4%) for integrated high‐risk HPV in cases exhibiting strong and diffuse ‘block‐type’ p16 expression.^3^

HPV‐independent VSCC comprise the majority of all VSCC in most developed countries.^4^ This group of patients has worse overall‐ and recurrence‐free survival, despite current treatments.^5^, ^6^ Therefore, special focus on the improvement of standard treatment in this particular group is warranted. Studies spanning the last two decades have reported that HPV‐independent VSCC are often driven by *TP53* mutations.^7^ Recent data, however, convincingly showed that a subset of HPV‐independent VSCC without *TP53* mutations were associated with an intermediate risk of recurrence.^6^ The existence of this third ‘histo‐molecular’ subclass is further supported by recent reports of HPV‐independent VSCC precursor lesions.^8^ Whether this VSCC subclass should be regarded as a distinct clinicopathological entity is currently still under debate.

In the meantime, it is necessary to develop a uniform approach towards the interpretation of p53 IHC patterns in vulvar cancer. Similar approaches in both endometrial and ovarian cancer have been succesful.^9^, ^10^, ^11^

A recent study categorised HPV‐independent VSCC based on p53 IHC as wild‐type or abnormal expression.^6^ The latter was associated with *TP53* mutations and consisted of diffuse strong nuclear overexpression or nuclear overexpression restricted to the basal layers of the tumour or complete absence of nuclear staining of tumour cells in the presence of a positive intrinsic control.^6^ In addition to these patterns, cytoplasmic p53 overexpression has been described as a fourth pattern which is associated with *TP53* mutations.^10^ Scattered and weak nuclear expressions were previously assigned as p53 IHC wild‐type.^6^ Finally, a pattern of nuclear p53 overexpression in which the basal keratinocytes were spared (exhibited no expression) has been described in HPV‐associated lesions.^12^ Although these six p53 IHC patterns have been recognised, their performance as surrogate marker for *TP53* mutational status has not been formally tested. Also, the interobserver agreement of this p53 IHC pattern‐based approach in VSCC is unknown. Therefore, we aimed to validate the performance of a pattern‐based p53 IHC interpretation in a large cohort of VSCC and assessed its reproducibility.

## Materials and methods

### CASE SELECTION

To constitute our cohort, we combined a retrospective case series of Leiden University Medical Center (LUMC, *n* = 48) and Vancouver General Hospital (*n* = 32) of patients who were surgically treated for primary invasive VSCC. For both centres, the cases were derived from larger cohorts which were previously tested for HPV presence by p16 IHC and HPV–polymerase chain reaction (PCR) and stained for p53 IHC.^6^, ^13^ In order to create a cohort in which all p53 IHC patterns were represented, one researcher [not involved in the p53 IHC scoring (KEK) enriched the cohort for HPV‐independent VSCCs including ‘uncommon’ p53 staining patterns. All FFPE blocks were cut into 4‐μm slides and stained with haematoxylin and eosin (H&E) and checked for the presence of VSCC by the local gynaecological pathologist. This study was granted ethical approval B16.024. Secondary use of tissue specimens adhered to the Dutch guidelines for proper use of human tissue.

### P53 IHC

For each case, p53 IHC was carried out locally with a protocol used for clinical purposes. Slides were stained with Dako Omnis and Dako EnVision^TM^ FLEX + detection system (p53 antibody, clone DO‐7, mouse monoclonal; Dako, Amstelveen, the Netherlands), although in a different solution (Vancouver 1:500 dilution, LUMC ready‐to‐use).

### PATTERN‐BASED P53 IHC SCORING

**Figure 1:**
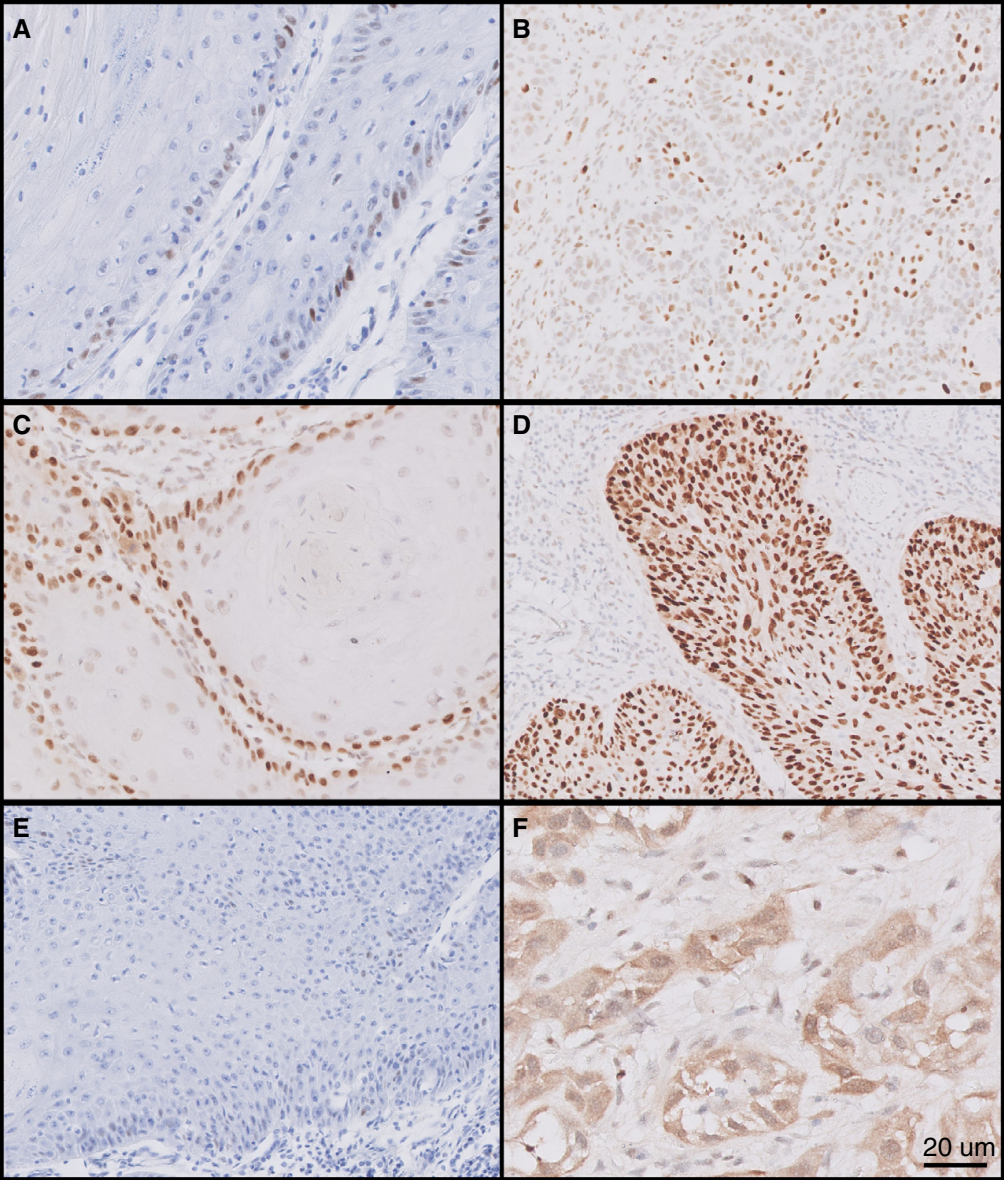
Six different p53 immunohistochemistry patterns in vulvar squamous cell carcinoma. **A,** Scattered p53 expression; **B,** mid‐epithelial p53 expression with notable sparing of the basal layer; **C,** basal expression; **D,** basal to parabasal/diffuse expression; **E,** absent p53 expression in the presence of an intrinsic positive control (either tumour cells or stromal cells); **F,** cytoplasmic expression. Scattered and mid‐epithelial (**A,B**) expression were designated under final p53 immunohistochemistry (IHC)‐class wild‐type, while the remaining four patterns (**C–F**) were designated under final p53 IHC‐class mutant. Scale bar 20 µm.

Based on the literature^11^ and experience from large retrospective cohorts, two p53 immunohistochemical staining patterns were considered to represent ‘final p53 IHC class wild‐type’ patterns; (1) scattered: heterogeneous nuclear staining of variable intensities in the basal and parabasal squamous tumour cells; and (2) mid‐epithelial: strong–moderate mid‐epithelial nuclear p53 expression of tumour cells, with notable basal sparing (this pattern is associated with the presence of HPV). The remaining four patterns were considered to represent ‘final p53 IHC class mutant’ patterns: (3) basal: strong nuclear expression of consecutive basal tumour cells (minor component of nuclear expression of parabasal tumour cells is acceptable); (4) basal to parabasal/diffuse: diffuse strong nuclear staining in basal and upper layers; (5) absent expression: complete absence of nuclear expression in the presence of an intrinsic positive control (stromal cells or adjacent normal epithelium); and (6) cytoplasmic: diffuse cytoplasmic staining with or without nuclear staining in the presence of a positive intrinsic control (Figure 1).^13^ All cases were scored for p53 IHC pattern and p53 IHC final class by two pathologists (T.B. and L.H.) independently.

Finally, cases with discordant interpretation of p53 IHC pattern were discussed between both observers in a consensus meeting, where both observers remained blinded to the NGS results (Supporting information, Figure 1).

### MUTATIONAL ANALYSIS

DNA was isolated locally from each case using different techniques. At the pathology department of LUMC, an area with> 70% tumour cells was annotated for microdissection. The total nucleic acids (DNA/RNA) were isolated from formalin‐fixed paraffin‐embedded (FFPE) tissue slides using a fully automated tissue preparation system (TPS) robot from Siemens Healthcare Diagnostics (Tarrytown, NY, USA).^14^ DNA was quantified with the Qubit fluorometric quantification system (Life Technologies, Gent, Belgium). Next‐generation sequencing (NGS) was performed with a customised Cancer Hotspot Panel (Life Technologies), covering the full exonic region of the *TP53* gene (exons 1–11), with a minimum read depth of 300. Sequencing analysis was performed on an Ion Torrent platform.

At the pathology department of Vancouver, DNA isolation was performed using a QiaAmp FFPE Tissue Kit (Qiagen, Toronto, ON, Canada). Samples were included when the base quality score was> 30. Moreover, the NGS panel covered exons 4–9 of the *TP53* gene, with a minimum read depth of 500. Sequencing analysis was performed on an Illumina Miseq platform.

NGS data analysis was manually executed by a blinded clinical molecular biologist (N.S.). *TP53* variants were assigned according to the five‐category classification: pathogenic, probably pathogenic, variant of unknown significance (VUS), probably benign and benign with a variant allele frequency (VAF) of at least 0.05.^15^ Only pathogenic and/or probably pathogenic *TP53* mutations were scored as *TP53* mutant.

### STATISTICAL ANALYSIS

For the data analysis and illustration of the graphs and figures, the statistical software package spss version 23.0 (SPSS Inc., Chicago, IL, USA) was used. The diagnostic test performance of p53 IHC patterns was quantified by Cohen's kappa for agreement, together with calculating the sensitivity and specificity accuracy of p53 IHC compared to *TP53* mutation status.

## Results

### CASE–SERIES

We started with a selection of 80 VSCC from our archives, five of which were HPV‐related and 75 HPV‐independent. p53 IHC was performed on all cases, three of which were excluded for further analysis due to the lack of intrinsic control (*n* = 2) or not adhering to the glass slide (*n* = 1). After NGS, 18 VSCCs were excluded due to insufficient quality and/or quantity of the DNA (VAF < 0.05 in the background with deamination artefacts). Finally, this resulted in a study cohort of 59 VSCCs (three HPV‐associated and 56 HPV‐independent) with interpretable results.

### REPRODUCIBILITY OF PATTERN‐BASED P53 IHC SCORING IN VSCC

The observers agreed in 79.7% (47 of 59) of the cases on p53 IHC patterns (Table 1), which was substantial (*k* = 0.71, *P* < 0.001). Twelve cases with disagreement on p53 IHC patterns were discussed in a consensus meeting (Supporting information, Table S1). Agreement was easily reached in one case due to a data entry error by one pathologist (case 10 in Supporting information, Table S1). In four of the remaining 11 cases, the observer's original interpretation differed between parabasal/diffuse (pattern 4) and basal p53 (pattern 3). Three of the 11 cases differed between scattered (pattern 1) and basal staining (pattern 3). In one of the 11 cases the original pattern score differed between mid‐epithelial (pattern 2) and parabasal/diffuse staining (pattern 4). Another case was scored scattered (pattern 1), but absent (pattern 5) by the other observer. Finally, two cases were scored cytoplasmic (pattern 6) by one observer, but parabasal and scattered by the other observer (Supporting information, Table S1). Agreement on p53 IHC patterns and thus final p53 IHC class interpretation was reached for all 12 discordant cases during the consensus meeting.

**Table 1:** p53 immunohistochemistry (IHC) patterns of 59 vulvar squamous cell carcinoma (VSCC) by two independent and blinded observers

		p53‐IHC patterns observer 1						
		Scattered	Mid‐epithelial with notable basal sapring	Basal	Basal and parabasal/diffuse	Absent	Cytoplasmic	Total
p53 IHC patterns observer 2	Scattered	13	0	0	0	0	0	13
	Mid‐epithelial with notable basal sparing	0	2	0	1	0	0	3
	Basal	3	0	1	3	0	0	7
	Basal and parabasal/diffuse	0	1	1	23	0	0	25
	Absent	1	0	0	0	5	0	6
	Cytoplasmic	1	0	0	1	0	3	5
	Total	18	3	2	28	5	3	59

### CONCORDANCE OF CONSENSUS P53 IHC PATTERNS WITH *TP53* MUTATIONAL STATUS

Of the 59 VSCCs, 43 VSCCs harboured *TP53* mutations and 16 were *TP53* wild‐type (Supporting information, Table S1). The concordance between the original final p53 IHC class and *TP53* mutation status was high for both observers independently (*k* = 0.76 and 0.91, *P* < 0.001, Table 2). This concordance increased to 0.92 [95% confidence interval (CI) = 0.81–1.00] when using the final p53 IHC class after the consensus meeting. The sensitivity and specificity of this approach were both high (95.3%, 95% CI = 82.9–99.1% and 100%, 95% CI = 75.9–100%, respectively). All final p53 IHC class mutant VSCC after consensus were *TP53* mutant. Two of the 59 VSCC that were assigned final p53 IHC class wild‐type were *TP53* mutant (Table 3).

**Table 2:** The p53 immunohistochemistry (IHC) patterns observed in vulvar squamous cell carcinoma (VSCC) in relation to the *TP53* mutation status before consensus

	Before consensus			
	Final p53 IHC class observer 1		Final p53 IHC class observer 2	
	Wild‐type p53 IHC	Mutant p53 IHC	Wild‐type p53 IHC	Mutant p53 IHC
TP53 wild‐type	15	1	15	1
TP53 mutant	5	38	1	42
Total	20	39	16	43

**Table 3:** The p53 immunohistochemistry (IHC) patterns observed in vulvar squamous cell carcinoma (VSCC) in relation to *TP53* mutation status after consensus

	After consensus		
	p53 IHC class wild‐type	p53 IHC class mutant	Total
TP53 wild‐type	16	0	16
TP53 mutant	2	41	43
Total	18	41	59
Sensitivity: 95.3% [95% confidence interval (CI) = 82.9–99.1%]			
Specificity: 100% (95% CI = 75.9–100%)			
Accuracy: 96.6%			

### TWO DISCORDANT CASES BETWEEN FINAL P53 IHC CLASS AND *TP53* MUTATIONAL STATUS

**Figure 2:**
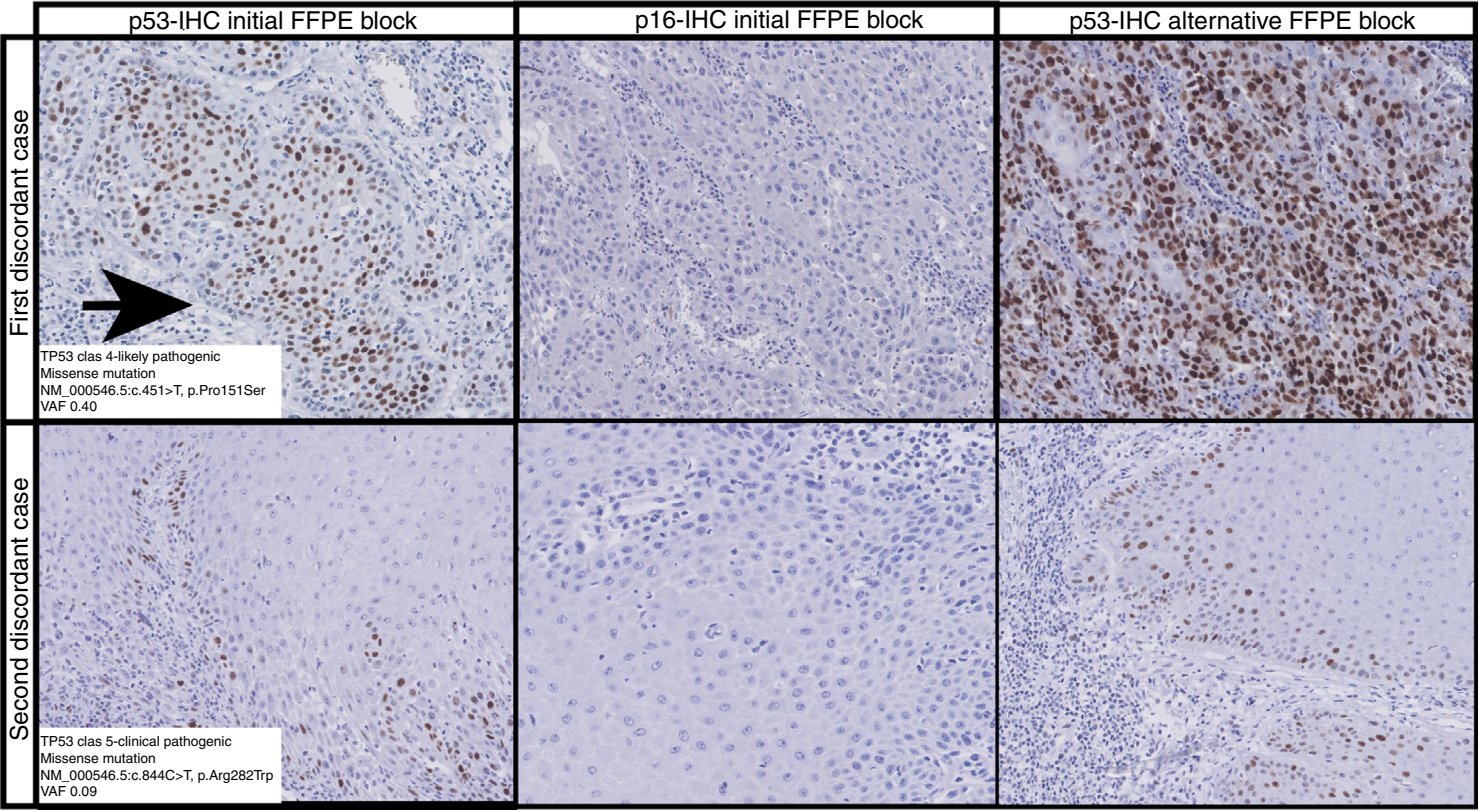
p53 immunohistochemistry of the two remaining discordant cases after consensus. NA, not applicable. For the first case (upper panel), consensus was reached on a mid‐epithelial p53 immunohistochemistry (IHC) expression pattern with basal sparing (indicated by arrow), while the tumour showed a pathogenic *TP53* mutation with a high variant allele frequency (VAF). An additional p16–IHC could prevent misinterpretation of the final p53 IHC class due to the absence of ‘block‐type’ p16 expression. Because of suboptimal staining, an alternative block of the same case was stained for p53 and a diffuse expression of p53 was observed. Moreover, after revising all haematoxylin and eosin (H&E) staining of this tumour revealed the presence of differentiated type, vulvar intra‐epithelial neoplasia (dVIN) as a precursor lesion, which increases the probability of harbouring *TP53* mutations. The second case (lower panel) was also human papillomavirus (HPV)‐unrelated, and scored scattered by both observers. We cannot explain the discordancy between the final p53 IHC class and mutational analysis of this case, although the VAF was low but reliable. Scale bar 20 µm.

The remaining two VSCC with a discordance between final p53 IHC class and *TP53* mutational status are shown in Figure 2. The first discordant case was a poorly differentiated HPV‐independent VSCC, and was originally scored as mid‐epithelial by one observer and parabasal by the other observer. After consensus, the observers agreed that despite suboptimal fixation and an attenuated basal layer, the staining represented a mid‐epithelial with notable sparing pattern (Figure 2). NGS of this case revealed a probably pathogenic *TP53* NM_000546.5: c.451C> T missense mutation (NP_000537.3:p.Arg282Trp) with a VAF of 0.4. To explore this in more detail we stained a slide from an alternative FFPE block of the same tumour for p53, in which a clear diffuse p53 overexpression (pattern 4) was observed (Figure 2). The second case was a well‐differentiated HPV‐independent VSCC and was not discussed at the consensus meeting, as both observers had interpreted the p53 IHC pattern as scattered (pattern 1). A p53 IHC of an alternative block also showed scattered p53 IHC. With NGS, we found a pathogenic *TP53* NM_000546.5: c.844C> T missense mutation (NP_000537.3:p.Pro151Ser) with a VAF of 0.09.

## Discussion

With this study, we have confirmed the use of p53 IHC patterns as an accurate predictor for the presence of *TP53* mutations in invasive VSCC as described by Tessier‐Cloutier *et al*.^13^ Moreover, we report a high interobserver reproducibility using this p53 IHC pattern based approach. The original p53 IHC pattern designation by the two observers resulted in an agreement of 80%, and when translated into a final p53 IHC class the accuracy was 97%.

### CASES WITH INITIAL DISAGREEMENT ON P53 IHC PATTERNS

Some of the p53 IHC pattern disagreements were attributable to a minor difference in the interpretation of pattern definitions. This was particularly true for the disagreement between ‘basal’ and ‘basal to parabasal/diffuse’ patterns (patterns 3 and 4), as the overexpression of p53 in the basal tumour cells often coincides with some level of parabasal overexpression. The lack of a defined threshold (how much parabasal expression is accepted for pattern 3) explains four cases for which there was original disagreement. During the consensus meeting, the observers therefore introduced an arbitrary rule;> 10% of the tumour should show parabasal overexpression to be assigned parabasal/diffuse (pattern 4). Hereafter, consensus was easily reached for all four cases, and the percentage of agreement on p53 IHC patterns increased to 88%. There were three cases where there was an initial disagreement between the basal‐overexpression pattern and scattered pattern, an area of difficulty already identified in the prior study by Tessier‐Cloutier *et al*.^13^ While all three cases were resolved on consensus review, in practice we acknowledge that a subset of these cases would need *TP53* mutation testing to differentiate the two. For this scenario (discriminating scattered basal versus basal overexpression) another arbitrary cut‐off to count consecutive p53 overexpressing cells may be considered. For p53 signatures in the fallopian tube, 12 consecutive p53 overexpressing cells have been proposed as a pragmatic approach to define *TP53* mutational status.^16^ Whether this threshold is applicable in the context of vulvar SCC with basal p53–IHC expression remains to be determined.

Despite a disagreement in p53 IHC pattern (pattern 3 versus pattern 4), the final p53 IHC class was not affected by this. In fact, independently of the minor differences in patterns scored, there was 90% agreement on final p53 IHC class prior to the consensus meeting. After the consensus, the accuracy to predict *TP53* mutations based on p53 IHC wild‐type/mutant staining was high (96.6%). This is comparable to ovarian and endometrial carcinomas, where p53 IHC serves as a robust surrogate marker for *TP53* mutations.^9^, ^11^

The mid‐epithelial pattern of p53 IHC expression (pattern 2) is one of the more unusual patterns and has only recently been described.^12^ It has been suggested that this pattern can be seen in HPV‐associated VSCC, as it represents senescence of tumour cells infected with persistent high‐risk HPV.^12^ In our previous study,^13^ this pattern was only observed in HPV‐associated VSCC and precancers.

### DISCORDANT CASES BETWEEN P53 IHC AND *TP53* MUTATION STATUS

We observed that recognising mid‐epithelial patterns can be challenging, and may be confused with either scattered wild‐type (pattern 1) or diffuse p53 overexpression (pattern 4). This is exemplified by case 1 in Figure 2, which was called mid‐epithelial upon consensus, but a second p53 IHC of an alternative block showed convincing diffuse overexpression. The addition of p16 IHC in cases in which the mid‐epithelial p53 IHC pattern is not so obvious might be useful, as pattern 2 is strongly associated with HPV‐related VSCC.^13^ In case 1, p16 IHC did not show ‘block‐type’ expression and HPV–PCR was negative. This would have helped in correct interpretation of the p53 IHC pattern and thus the final p53 IHC class. Thereby, this example supports the interpretation of p53 staining in VSCC in conjunction with p16 IHC.

The second discordant case showed a scattered p53 IHC pattern (agreed upon by both observers for which no consensus was needed), but bared a pathogenic *TP53* mutation (Figure 2). The tumour tissue appeared well fixed, and a review of all H&E slides of this case did not show obvious precancerous lesions such as dVIN or HSIL in the adjacent surface epithelium. The margins, however, showed epithelial changes that are in line with the spectrum of changes described as ‘verrucous acanthosis with altered differentiation (VAAD)’. In conformity, this precursor lesion also showed a weak and scattered p53 IHC pattern. Revision of the raw NGS data confirmed low background noise, which strengthens the finding that this particular mutation is a true mutation and not a deamination artefact, despite the low VAF. A possible explanation for this discordance could be an early emerging *TP53* mutant clone which was picked up by the NGS, but was not detected by the p53 IHC. As this patient was diagnosed with VSCC in 2012 and did not develop recurrences, we were unable to confirm this hypothesis.

This study has some limitations, one being the composition and size of our study cohort. The cohort was relatively small and enriched for unusual p53 IHC patterns, and therefore the distribution of the observed p53 IHC patterns remains unknown. We therefore encourage subsequent studies to use larger and unselected VSCC cohorts in order to study these p53 IHC patterns and validate our findings. The use of tissue microarray approaches are discouraged for this, as some of these p53 IHC patterns require a good overview of the tumour.

In conclusion, this paper is the first, to our knowledge, to test the performance of the newly proposed p53 IHC pattern based interpretation in VSCC. We show that p53 IHC pattern interpretation is highly reproducible, and can serve as a reliable surrogate approach for assigning final p53 IHC class. We would like to emphasise that the high agreement was achieved in the context of optimal laboratory protocols with adequate controls (recommended external on slide control tissue; tonsil^10^) and review by specialised pathologists. Experience, training and proper p53 IHC staining protocols are required in order to translate our findings into routine diagnostic pathology. Nonetheless, this study represents a solid basis for further characterising the clinical relevance of stratifying (HPV‐independent) VSCC based on final p53 IHC class.

## Conflict of interest

None.

## Author contributions

Conception and design: KEK, LNH, TB. Acquisition of data: KEK, NSW, TAR, LNH, TB Analysis and Interpretation of data: KEK, NSW, LNH, TB. Writing and review of the manuscript: KEK, NSW, BTC, MIEvP, CBG, LNH, TB. Study supervision: TB.

## Supporting information


**Figure S1** **.** Study design

**Table S1.** Information on p53 immunohistochemistry patterns and *TP53* mutations of all cases in the study cohort.
