# Supplementary figures and images for: Performance of the pattern‐based interpretation of p53 immunohistochemistry as a surrogate for TP53 mutations in vulvar squamous cell carcinoma
Source: Histopathology. 2020 Jun 7;77(1):92–9. doi: 10.1111/his.14109 (PMC7383647; doi:10.1111/his.14109)

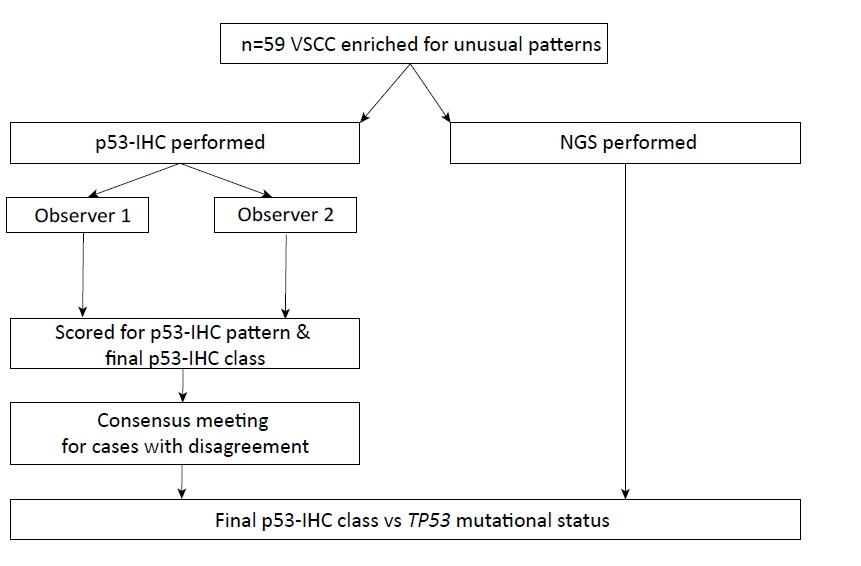

Supplement: Supplementary file 1 — Figure S1 . Study design [file HIS-77-92-s001.jpg]
