# Supplementary material for: Performance of the pattern‐based interpretation of p53 immunohistochemistry as a surrogate for TP53 mutations in vulvar squamous cell carcinoma
Source: Histopathology. 2020 Jun 7;77(1):92–9. doi: 10.1111/his.14109 (PMC7383647; doi:10.1111/his.14109)
Supplement: Supplementary file 2 — Table S1. Information on p53 immunohistochemistry patterns and TP53 mutations of all cases in the study cohort. [file HIS-77-92-s002.docx]

**Supplemental table 1. Information on p53 immunohistochemistry patterns and *TP53* mutations of all cases in the validation cohort.**

|  | **Immunohistochemistry** | | | | | | | | | **Next-generation sequence** | | | | |
| --- | --- | --- | --- | --- | --- | --- | --- | --- | --- | --- | --- | --- | --- | --- |
|  | **Case** | **Histologic grade** | **p16-IHC** | **P53-IHC Pattern Observer 1** | **Final P53-IHC class Observer 1** | **P53-IHC Pattern Observer 2** | **Final P53-IHC class Observer 2** | **P53-IHC pattern Consensus** | **Final p53-IHC class Consensus** | ***TP53*** | **VAF** | **Chromosome** | **HGVS coding** | **Mutation type** |
| **Disagreement on P53-IHC patterns** | 1 | moderately | neg | Parabasal /diffuse | mutant | basal | mutant | parabasal/ diffuse | mutant | mutant | 0.14 | 17:7577120 | NM_000546.5:c.818G>A | missense |
|  | 2 | moderately | neg | scattered | mutant | basal | mutant | parabasal/ diffuse | mutant | mutant | 0.31 | 17:7578406 | NM_000546.5:c.524G>A | missense |
|  | 3 | poorly | neg | mid-epithelial | wildtype | parabasal/ diffuse | mutant | mid-epithelial | wildtype | mutant | 0.40 | 17:7578479 | NM_000546.5:c.451C>T | missense |
|  | 4 | moderately | neg | scattered | wildtype | basal | mutant | basal | mutant | mutant | 0.12 | 17:7577534 | NM_000546.5:c.747G>T NM_000546.5:c.586C>T | missense |
|  |  |  |  |  |  |  |  |  |  |  | 0.17 | 17:7578263 |  | nonsense |
|  | 5 | poorly | neg | Parabasal /diffuse | mutant | basal | mutant | basal | mutant | mutant | 0.13 0.29 | 17:7577114 17:7578413 | NM_000546.5:c.824G>T  NM_000546.5:c.517G>T | missense missense |
|  | 6 | moderately | neg | scattered | wildtype | basal | mutant | scattered | wildtype | wildtype |  |  |  |  |
|  | 7 | poorly | neg | Parabasal /diffuse | mutant | basal | mutant | basal | mutant | mutant | 0.16 |  | NM_000546.5:c.824G>A | missense |
|  | 8 | poorly | neg | basal | mutant | parabasal/ diffuse | mutant | Parabasal/ diffuse | mutant | mutant | 0.28 0.21 |  | NM_000546.5c.524G>A NM_000546.5c.430C>T | missense nonsense |
|  | 9 | well | neg | scattered | wildtype | absent | mutant | absent | mutant | mutant | 0.35 |  | NM_000546.5c.193A>T | nonesense |
|  | 10 | moderately | pos | Parabasal /diffuse | mutant | mid-epithelial | wildtype | mid-epithelial | wildtype | wildtype |  |  |  |  |
|  | 11 | moderately | neg | Parabasal /diffuse | mutant | cytoplasmic | mutant | cyoplasmic | mutant | mutant | 0.17 |  | NM_000546.5c.404G>T | missense |
|  | 12 | well | neg | scattered | wildtype | cytoplasmic | mutant | cytoplasmic | mutant | mutant | 0.20 |  | NM_000546.5c.920-1G>A | splice-site |
| **Agreement on p53-IHC patterns** | 13 | poorly | neg | Parabasal /diffuse | mutant | parabasal/ diffuse | mutant |  |  | mutant | 0.42 | 17:7578266 | NM_000546.5:c.583A>T | missense |
|  | 14 | well | neg | scattered | wildtype | scattered | wildtype |  |  | mutant | 0.09 | 17:7577094 | NM_000546.5:c.844C>T | missense |
|  | 15 | poorly | neg | Parabasal /diffuse | mutant | parabasal/ diffuse | mutant |  |  | mutant | 0.20 | 17:7578526 | NM_000546.5:c.404G>T | missense |
|  | 16 | poorly | neg | Parabasal /diffuse | mutant | parabasal/ diffuse | mutant |  |  | mutant | 0.43 | 17:7577538 | NM_000546.5:c.743G>A | missense |
|  | 17 | poorly | neg | Parabasal /diffuse | mutant | parabasal/ diffuse | mutant |  |  | mutant | 0.32 | 17:7577565 | NM_000546.5:c.716A>G | missense |
|  | 18 | poorly | neg | absent | mutant | absent | mutant |  |  | mutant | 0.19 | 17:7579723 | NM_000546.5:c.75-2A>T | splice-site |
|  | 19 | poorly | neg | absent | mutant | absent | mutant |  |  | mutant | 0.29 | 17:7578402 | NM_000546.5:c.528C>A | nonsense |
|  | 20 | poorly | neg | Parabasal /diffuse | mutant | parabasal/ diffuse | mutant |  |  | mutant | 0.48 | 17:7578461 | NM_000546.5:c.469G>T | missense |
|  | 21 | well | neg | mid-epithelial | wildtype | mid-epithelial | wildtype |  |  | wildtype |  |  |  |  |
|  | 22 | moderately | neg | cytoplasmic | mutant | cytoplasmic | mutant |  |  | mutant | 0.34 | 17:7576855 | NM_000546.5:c.991C>T | nonsense |
|  | 23 | moderately | pos | Parabasal /diffuse | mutant | parabasal/ diffuse | mutant |  |  | mutant | 0.41 | 17:7578203 | NM_000546.5:c.646G>C | missense |
|  | 24 | poorly | neg | Parabasal /diffuse | mutant | parabasal/ diffuse | mutant |  |  | mutant | 0.13 | 17:7577543 | NM_000546.5:c.734_738delGCATG | frame-shift |
|  | 25 | well | pos | mid-epithelial | wildtype | mid-epithelial | wildtype |  |  | wildtype |  |  |  |  |
|  | 26 | poorly | neg | Parabasal /diffuse | mutant | parabasal/ diffuse | mutant |  |  | mutant | 0.46 | 17:7577571 | NM_000546.5:c.710delT | frame-shift |
|  | 27 | poorly | neg | Parabasal /diffuse | mutant | parabasal/ diffuse | mutant |  |  | mutant | 0.29 0.15 | 17:7578413 17:7577114 | NM_000546.5:c.517G>T NM_000546.5:c.824G>T | missense missense |
|  | 28 | well | neg | scattered | wildtype | scattered | wildtype |  |  | wildtype |  |  |  |  |
|  | 29 | poorly |  | parabasal/ diffuse | mutant | parabasal/ diffuse | mutant |  |  | mutant |  |  | NM_000546.5: c.517G>T | missense |
|  | 30 | poorly | neg | parabasal/ diffuse | mutant | parabasal/ diffuse | mutant |  |  | mutant |  |  | NM_000546,5 c.818G>A | missense |
|  | 31 | poorly | neg | parabasal/ diffuse | mutant | parabasal/ diffuse | mutant |  |  | mutant | 0.30 | 17:7579361 | NM_000546.5:c.326T>C | missense |
|  | 32 | moderately | neg | Cytoplasmic (basal) | mutant | cytoplasmic | mutant |  |  | mutant | 0.51 | 17:7576855 | NM_000546.5:c.991C>T | nonsense |
|  | 33 | poorly | neg | parabasal/ diffuse | mutant | parabasal/ diffuse | mutant |  |  | mutant | 0.33 0.34 |  | NM_000546.5c.839G>C NM_000546.5c.524delG | missense frameshift |
|  | 34 | well | neg | scattered | wildtype | scattered | wildtype |  |  | wildtype |  |  |  |  |
|  | 35 | poorly | neg | parabasal/ diffuse | mutant | parabasal/diffuse | mutant |  |  | mutant | 0.19 |  | NM_000546.5c.404G>T | missense |
|  | 36 | well | neg | scattered | wildtype | scattered | wildtype |  |  | wildtype |  |  |  |  |
|  | 37 | poorly | neg | scattered | wildtype | scattered | wildtype |  |  | wildtype |  |  |  |  |
|  | 38 | moderately | neg | scattered | wildtype | scattered | wildtype |  |  | wildtype |  |  |  |  |
|  | 39 | poorly | neg | parabasal/ diffuse | mutant | parabasal/ diffuse | mutant |  |  | mutant | 0.49 |  | NM_000546.5c.733G>A | missense |
|  | 40 | well | neg | scattered | wildtype | scattered | wildtype |  |  | wildtype |  |  |  |  |
|  | 41 | poorly | neg | parabasal/ diffuse | mutant | parabasal/ diffuse | mutant |  |  | mutant | 0.65 |  | NM_000546.5c.817C>G | missense |
|  | 42 | poorly | neg | absent | mutant | absent | mutant |  |  | mutant | 0.18 |  | NM_000546.5c.763_766dupATCA | frameshift |
|  | 43 | poorly | neg | parabasal/ diffuse | mutant | parabasal/ diffuse | mutant |  |  | mutant | 0.45 |  | NM_000546.5c.481G>A | missense |
|  | 44 | well | neg | scattered | wildtype | scattered | wildtype |  |  | wildtype |  |  |  |  |
|  | 45 | well | neg | scattered | wildtype | scattered | wildtype |  |  | wildtype |  |  |  |  |
|  | 46 | moderately | neg | parabasal/ diffuse | mutant | parabasal/ diffuse | mutant |  |  | mutant | 0.07 |  | NM_000546.5c.742C>T | missense |
|  | 47 | poorly | neg | absent | mutant | absent | mutant |  |  | mutant | 0.15 |  | NM_000546.5c.880G>T | nonesense |
|  | 48 | well | neg | scattered | wildtype | scattered | wildtype |  |  | wildtype |  |  |  |  |
|  | 49 | poorly | neg | absent | mutant | absent | mutant |  |  | mutant | 0.11 |  | NM_000546.5c.763_766dupATCA | frameshift |
|  | 50 | moderately | neg | parabasal/ diffuse | mutant | parabasal/ diffuse | mutant |  |  | mutant | 0.32 |  | NM_000546.5c.817C>G | missense |
|  | 51 | moderately | neg | parabasal/ diffuse | mutant | parabasal/ diffuse | mutant |  |  | mutant | 0.18 |  | NM_000546.5c.711G>A | missense |
|  | 52 | poorly | neg | basal | mutant | basal | mutant |  |  | mutant | 0.26 |  | NM_000546.5c.713G>C | missense |
|  | 53 | poorly | neg | parabasal/ diffuse | mutant | parabasal/ diffuse | mutant |  |  | mutant | 0.77 |  | NM_000546.5c.844C>T | missense |
|  | 54 | well | neg | scattered | wildtype | scattered | wildtype |  |  | wildtype |  |  |  |  |
|  | 55 | moderately | neg | scattered | wildtype | scattered | wildtype |  |  | wildtype |  |  |  |  |
|  | 56 | poorly | neg | parabasal/ diffuse | mutant | parabasal/ diffuse | mutant |  |  | mutant | 0.77 |  | NM_000546.5c.818G>A | missense |
|  | 57 | moderately | neg | scattered | wildtype | scattered | wildtype |  |  | wildtype |  |  |  |  |
|  | 58 | poorly | neg | parabasal/ diffuse | mutant | parabasal/ diffuse | mutant |  |  | mutant | 0.57 |  | NM_000546.5c.817C>T | missense |
|  | 59 | poorly | neg | cytoplasmic | mutant | cytoplasmic | mutant |  |  | mutant | 0.88 |  | NM_000546.5c.780delC | frameshift |
